# In vitro invasion efficiency and intracellular proliferation rate comprise virulence-related phenotypic traits of *Neospora caninum*

**DOI:** 10.1186/1297-9716-42-41

**Published:** 2011-02-23

**Authors:** Javier Regidor-Cerrillo, Mercedes Gómez-Bautista, Itsaso Sodupe, Gorka Aduriz, Gema Álvarez-García, Itziar Del Pozo, Luis Miguel Ortega-Mora

**Affiliations:** 1 SALUVET, Animal Health Department, Complutense University of Madrid, Ciudad Universitaria s/n, 28040 Madrid, Spain; 2 NEIKER-Tecnalia, Berreaga 1, Derio, 48160 Vizcaya, Spain

## Abstract

In this study, we examined the in vitro invasion and proliferation capacities of the Nc-Liv and ten Spanish *Neospora caninum *isolates (Nc-Spain 1 H - Nc-Spain 10). The invasion rate was determined as the number of tachyzoites that completed their internalisation into MARC-145 cells at 2, 4, and 6 h post-inoculation (pi). The proliferation rate was evaluated by determining the doubling time during the exponential proliferation period. Significant differences in the invasion rates of these isolates were detected at 2 and 4 h pi (*P *< 0.0001, Kruskal-Wallis test). At 4 h pi, the Nc-Spain 4 H and Nc-Liv isolates displayed the highest, while the Nc-Spain 3 H and Nc-Spain 1 H isolates had the lowest invasion rates (by Dunn's test). Variations in the proliferation kinetics of these isolates were also observed. Between different isolates, the lag phase, which occurs before the exponential growth phase, ranged from 8 to 44 h, and the doubling time ranged from 9.8 to 14.1 h (*P *= 0.0016, ANOVA test). Tachyzoite yield, which combines invasion and proliferation data, was also assessed and confirmed marked differences between the highly and less prolific isolates. Interestingly, a direct correlation between the invasion rates and tachyzoite yields, and the severity of the disease that was exhibited by infected pregnant mice in previous works could be established for the isolates in this study (Spearman's coefficient > 0.62, *P *< 0.05). The results of this study may help us to explain the differences in the pathogenicity that are displayed by different isolates.

## Introduction

*Neospora caninum *is an obligate intracellular parasite that is phylogenetically related to *Toxoplasma gondii *and causes neuromuscular disease in dogs and abortion in cattle, although it can infect other host species [1,2]. Neosporosis is currently recognised as one of the main causes of infectious bovine abortion worldwide [1].

Previous studies have demonstrated that differences occur in the genetic and biological characteristics of *N. caninum *isolates. Thus, genetic diversity among *N. caninum *isolates has been detected using different polymorphic markers [3], including those that are based on microsatellite sequences, which were demonstrated to be the most suitable for typing *N. caninum *isolates [4-8]. Importantly, *N. caninum *isolates exhibit differences in their capacity to produce pathology in cerebral mouse models [9-12], and in their efficacy to be transmitted from dams to offspring [13-16]. Genetic and biological intra-specific diversity of *N. caninum *isolates may influence their capacity to produce disease in the natural host, and the clinical presentation and epidemiology of neosporosis.

Very little information is known about the inherent factors of this parasite that contribute to its intra-specific pathogenicity, but the capacity to produce pathology has been associated with the behaviour of different *N. caninum *isolates in the host. The dissemination capacity, the parasite burdens that are reached in target tissues, the ability to avoid the immune response produced against the infection by the host and the rate of tachyzoite-bradyzoite conversion in the host may all contribute to the different levels of pathogenicity that are caused by different isolates [11-13,16,17]. Previous in vitro studies have reported that the growth [18,19] and bradyzoite conversion rates [14,20,21] are variable among different *N. caninum *isolates. Additionally, the low pathogenicity levels of the Nc-Spain 1 H isolate in mice and cattle have been attributed to the low viability rate and tachyzoite yield of this isolate in cell cultures [14,15]. Therefore, similar to *T. gondii*, the inherent pathogenicity of different *N. caninum *isolates may be directly related to specific virulence traits, which include the migration capacity, the ability to cross barriers and the cell invasion and intracellular proliferation efficiencies [22-25].

In this work, we investigated the association between the in vitro phenotypes that were displayed by *N. caninum *isolates and their pathogenicity. Specifically, we examined the invasion efficiencies and the intracellular proliferation kinetics of eleven *N. caninum *isolates, including the naturally attenuated NcSpain-1 H isolate and the highly pathogenic Nc-Liverpool isolate, which showed profound differences in their vertical transmission characteristics and their capacities to induce pathology in pregnant cattle [14,26].

## Materials and methods

### Cell cultures, parasites and preparation of *N. caninum *isolates for in vitro assays

The *N. caninum *isolates that were used in this study are shown in Table 1. The Spanish *N. caninum *isolates and the Nc-Liverpool (Nc-Liv) isolate were routinely maintained in a monolayer culture of the MARC-145 monkey kidney cell line after reactivation from cryovials, as described previously [5]. The Nc-Liv isolate was previously passaged in a mouse and re-isolated in MARC-145 cell cultures as described previously [16], to minimise the occurrence of potential alterations in its biological characteristics due to prolonged cell culture maintenance, as has been previously reported [27]. The *N. caninum *isolates that were used in these in vitro assays were subjected to a limited number of culture passages (Table 1).

**Table 1:** *Neospora caninum *isolates included in the in vitro assays.

Isolate*	Host origin**	Geographical origin^&^	Passages number^§^	Neonatal morbidity (%)^#^	Neonatal mortality (%)^#^	Vertical transmission rate (%)^#^
Nc-Spain 1H^a^	2-day-old healthy calf	Madrid	12-18	0^L^	5^L^	5^L^
Nc-Spain 2H	2-day-old healthy calf	Zaragoza	7-13	46.1^L^	20.4^L^	61.3^L^
Nc-Spain 3H^b^	52-day-old healthy calf	Navarra^1^	19-25	10.6^L^	7.7^L^	89^H^
Nc-Spain 4H^b^	22-day-old healthy calf	Navarra^1^	12-18	100^H^	100^H^	97.3^H^
Nc-Spain 5H	14-day-old healthy calf	León	12-18	98.6^H^	96^H^	100^H^
Nc-Spain 6	30-day-old healthy calf	País Vasco	20-26	34.5^L^	29.8^L^	57.6^L^
Nc-Spain 7	57-day-old healthy calf	Navarra^2^	17-23	98.3^H^	95^H^	79.1^L^
Nc-Spain 8	2-day-old healthy calf	Navarra^1^	8-14	4.7^L^	1.1^L^	56.4^L^
Nc-Spain 9	7-day-old healthy calf	Navarra^2^	9-15	39^L^	32.5^L^	52.6^L^
Nc-Spain 10^a^	2-day-old affected calf?	Madrid	21-27	25.5^L^	17.9^L^	65.5^L^
Nc-Liverpool	4-week-old affected dog	UK	12R-19R^@^	100^H^	100^H^	95.6^H^

Summary of name, genetic characterisation, host and geographical origin, passage number in cell culture, and their pathogenicity in a BALB/c pregnant mouse model determined in previous studies [14,16].

* Nc-Liv and Spanish isolates were genetically characterised by microsatellite analysis. Letters in superscript (^a^) and (^b^) indicate isolates with identical microsatellite profiles [4,5,14].

**All Spanish isolates were obtained from asymptomatic calves from different cattle. Nc-Spain 10 was isolated from an affected calf, but its clinical signs could not be attributed to *Neospora *infection [5,14]. Nc-Liv was isolated from a clinically affected dog [50].

**^&^**The geographical origin of Spanish isolates is indicated by province. Calves from Navarra originated from two dairy herds. Numbers in superscript (^1^) and (^2^) identify the dairy herd. Nc-Liverpool was isolated in the United Kingdom.

^§^The total number of cell culture passages of the *N. caninum *isolates included in these in vitro assays. (^@^) marks the total passages after re-isolation of Nc-Liv in cell culture from BALB/c *nu/nu *mice.

**^#^**The percentages of neonatal morbidity and neonatal mortality and vertical transmission rates were determined in previous studies using a pregnant BALB/c mouse model [14,16]. ^L, H ^Isolates were categorised into lowly/moderately pathogenic (^L^) or highly pathogenic (^H^) groups according to the significant differences found in neonatal morbidity and mortality. Identical superscript letters denote highly (^H^) and lowly/moderately transmissible (^L^) isolates.

The tachyzoites that were used in the in vitro assays were recovered from 3.5-day growth cultures, when the majority of the parasites were still intracellular (at least 80% of the parasite vacuoles were undisrupted in the cell monolayer), and purified using PD-10 (Sephadex G-25) columns (GE-Healthcare, Buckinghamshire, UK) prior to cell monolayer inoculations [28]. The tachyzoite inoculation dose was previously optimised for the maintenance of each isolate to estimate the recovery of tachyzoites from infected cultures under optimal conditions (actively replicating) at 3.5 days post-inoculation (pi). Infected cell cultures were scraped with a plastic cell scraper, harvested by centrifugation at 1 350 *g *for 10 min and suspended in Dulbecco Minimum Essential Medium (DMEM) supplemented with a 2% antibiotic-antimycotic solution (Gibco BRL, Paisley, UK), 10 mM HEPES and 2% heat-inactivated foetal bovine serum (FBS). The FBS employed in all in vitro assays was from the same production batch. Disrupted cell cultures were passed through 25 gauge needles, and tachyzoites were purified with PD-10 columns that had been previously equilibrated with the medium mentioned above. Tachyzoites were eluted from the columns with 5 mL of medium, and the number of tachyzoites was determined by trypan blue exclusion followed by counting in a Neubauer chamber. Tachyzoites were then resuspended at the required doses (2 × 10^5 ^tachyzoites/mL). Tachyzoite purification was performed at 4°C, and MARC-145 monolayers were inoculated within one hour of tachyzoite collection from flasks.

### In vitro invasion assays

Host cell invasion was measured using a double (red/green) immunostaining probe that was described previously [28] and a laser scanning-cytometer-based assay that was previously described for *T. gondii *[29], with several modifications. All of the isolates were assayed in triplicate, and all of the assays were performed in three independent experiments. The Nc-Liv isolate was included as a control in each batch of experiments. Additionally, MARC-145 monolayers that were not inoculated were immunostained and included as negative controls in each assay.

#### Double immunofluorescence staining

Purified parasites (2 × 10^5 ^tachyzoites) were added onto MARC-145 monolayers that had grown to confluence on circular (13 mm diameter) glass coverslips and incubated at 37°C in a 5% CO_2 _humidified incubator. At specific time periods (2 h, 4 h and 6 h pi), the coverslips were washed three times with 1 mL of phosphate buffered saline (PBS), fixed for 15 min in a 3% formaldehyde/0.05% glutaraldehyde solution and blocked with 3% bovine serum albumin (BSA) (Sigma-Aldrich, St. Louis, MO, USA) in PBS for 30 min. After blocking, the samples were labelled for 1 h with a 1:4000 dilution of a hyperimmune rabbit antiserum that was directed against *N. caninum *tachyzoites in PBS/0.3% BSA, washed three times with PBS and then labelled for 1 h with a 1:100 dilution of a secondary goat anti-rabbit IgG that was conjugated to PE-Cy5.5 (*red*, Invitrogen, Carlsbad, CA, USA). After washing, the samples were permeabilised with 0.25% Triton X-100 and blocked with PBS/3% BSA for 30 min. Parasites were then labelled with the rabbit anti-tachyzoite serum as described above, washed, and labelled with a 1:1000 dilution of a secondary goat anti-rabbit IgG conjugated to Alexa Fluor 488 (*green*, Molecular Probes, Eugene, OR, USA). Finally, the coverslips were washed, mounted on slides embedded with a 40% glycerol/2.5% 1,4-diazabicyclo[2.2.2]octane (Sigma-Aldrich) solution in PBS and analysed using a laser scanning cytometer. Polyclonal rabbit anti-*N. caninum *antiserum was raised in female New Zealand White rabbits (Harland Interfauna S.A., Barcelona, Spain) as described [30].

#### Laser scanning cytometry

The coverslips were analysed on a CompuCyte laser scanning cytometer LSC (CompuCyte, Cambridge, MA, USA) equipped with a BX50 upright fluorescence microscope (Olympus America, Melville, NY, USA). A 60.8 mm^2 ^(4.4 mm radius) circular area was scanned with a 20× objective, an argon ion excitation laser (488 nm), and two detection filters (530/30 (green) and 650 LP (red)). Data were acquired and analysed using Wincyte 3.4 software (CompuCyte).

#### Invasion rate determination

The invasion rate (IR) was determined to be the number of green events (extracellular and intracellular parasites) minus the number of red events (extracellular parasites) per scanned field at specific times (2 h, 4 h and 6 h pi). Negative controls (the MARC-145 monolayer that was not inoculated) were included to eliminate potential fluorescent artefacts. No events were observed in the negative control samples.

### In vitro intracellular proliferation assays

Proliferation kinetics were determined by quantifying the number of tachyzoites at specific times (4 h, 8 h, 20 h, 32 h, 44 h, 56 h, and 68 h pi) by real-time PCR (qPCR). All of the isolates were assayed in triplicate, and all of the assays were performed in three independent experiments. The Nc-Liv isolate was included in each batch of experiments as mentioned above, and MARC-145 monolayers that were not inoculated were used as negative controls for PCR analyses.

#### Culture conditions

MARC-145 cells were grown to confluency in 24-well tissue culture plates. Purified *N. caninum *parasites (2 × 10^5 ^tachyzoites) were added to the monolayers at time 0 and incubated for 4 h at 37°C in 5% CO_2_. Next, the non-invading parasites were removed by washing the monolayer three times with DMEM/2% heat-inactivated FBS/2% antibiotic solution. The cultures were subsequently maintained at 37°C in 5% CO_2 _for the time periods mentioned above. The samples were visualised by light microscopy to monitor parasite proliferation prior to their collection. Then, the media were removed and the cell cultures were recovered in 200 μL of PBS, 180 μL of lysis buffer and 20 μL of proteinase K (Qiagen, Hilden, Germany). The samples were transferred to Eppendorf tubes and were frozen at -80°C prior to DNA extraction.

#### DNA extraction and real-time PCR

Genomic DNA was extracted from cellular samples using the BioSprint 96 workstation and the BioSprint 96 DNA blood kit (Qiagen) according to the manufacturer's instructions. Genomic DNA was eluted in a final volume of 100 μL.

Quantification of *N. caninum *DNA was performed by real-time PCR targeting the Nc-5 region as described previously [31]. A total of 5 μL (100 ng) DNA was used for the PCR amplifications. The number of *N. caninum *parasites was calculated by interpolating the corresponding Ct values (cycle threshold value, which represents the fractional cycle number reflecting a positive PCR result) on a standard curve that was generated in each real-time PCR run by assaying 10-fold serial dilutions of parasite DNA, which was equivalent to 10^-1^-10^5 ^tachyzoites. All of the tachyzoite quantifications were assessed from average values obtained from duplicate determinations. DNA extracted from the uninfected MARC-145 monolayer was also included as a negative PCR control in each batch of reactions.

#### Proliferation rate, doubling time and tachyzoite yield determinations

The proliferation rate (μ) and doubling time (Td) were assessed for each assay during the exponential multiplication period by applying non-linear regression analysis and an exponential growth equation using GraphPad Prism 5 Demo, v. 5.00 software (GraphPad, San Diego, CA, USA). The μ and Td for each isolate were defined as the average value obtained from all of the determinations that revealed a linear regression, *R^2 ^*≥ 0.95.

The tachyzoite yield (TY_56h_) was defined as the average value of the number of tachyzoites quantified by qPCR at 56 h pi.

### Data statistics and correlation analysis

Differences between IRs determined over successive time points for each isolate were compared using the U Mann-Whitney test (2 h versus 4 h, 2 h versus 6 h, 4 h versus 6 h). The Kruskal-Wallis test was employed for comparisons among the IRs shown for the different isolates within each time point (2 h, 4 h, and 6 h pi). When statistically significant differences were found with the Kruskal-Wallis test, a Dunn's multiple-comparison test was applied to examine all of the possible pair-wise comparisons. A one-way ANOVA test, followed by the Tukey's multiple range tests, was employed to compare the Tds and TY_56h_s assessed for each isolate. Statistical analyses were carried out using a dataset composed of the values determined for each replicate obtained from the three independent experiments. The significance for these analyses was established at *P *< 0.05.

The Spearman's rank correlation coefficient (ρ) was applied to investigate the potential association between the in vitro parameters evaluated in this study (IR_2h_, IR_4h_, IR_6h_, Td, TY_56h_) and the neonatal morbidity, mortality and vertical transmission rates induced by these isolates in a well-established pregnant mouse model (Table 1) [14,16].

Statistical and correlation analyses were performed, and graphics were generated using GraphPad Prism 5 Demo, v. 5.00 software (GraphPad).

## Results

### Invasion rate comparisons

The IRs (the median number of intracellular events) of almost all isolates significantly increased from 2 h to 4 h pi or from 2 h to 6 h pi (*P *< 0.05, *U *Mann-Whitney test), with the exception of the Nc-Spain 9 isolate. However, no significant differences were found between the IRs of most of the isolates at 4 and 6 h pi. The IRs were only observed to significantly increase from 4 h to 6 h pi for the Nc-Spain 1 H and Nc-Spain 10 isolates.

**Figure 1:**
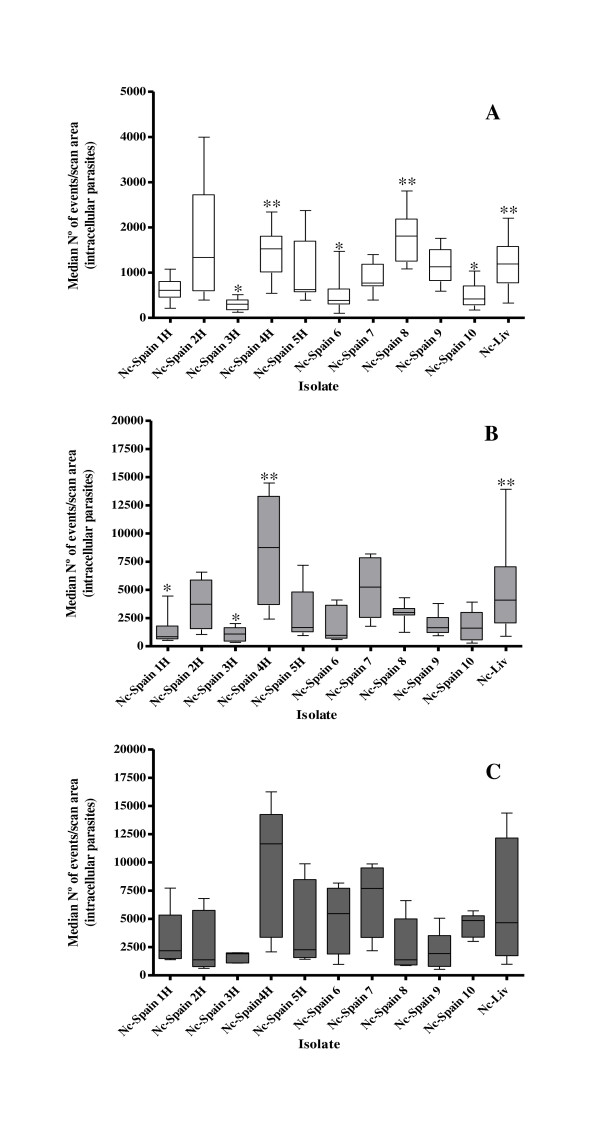
**Box-plot graphs representing the maximum and minimum values, lower and upper quartiles and medians of invasion rate (IR) replicates from experiments performed in triplicate determined in vitro for each *N. caninum *isolate**. IRs at 2 h pi (A). IRs at 4 h pi (B). IRs at 6 h pi (C). Error bars indicate the SD. (**) marks the significantly higher IRs compared with all of those IRs that were significantly lower (*) according to the Kruskal-Wallis test and the Dunn's multiple-comparison test.

Significant differences were also found among the IRs of different isolates at 2 and 4 h pi (*P *< 0.0001, Kruskal-Wallis test) (Figure 1A and B). At 2 h pi, the Nc-Spain 4 H, Nc-Spain 8 and Nc-Liv isolates exhibited the highest IRs (IR_2h_) in comparison to the IR_2h_s of the Nc-Spain 3 H, Nc-Spain 6 and Nc-Spain 10 isolates, which displayed the lowest values (by Dunn's test). At 4 h pi, the Nc-Spain 4 H and Nc-Liv isolates exhibited significantly higher IRs (IR_4h_) than the Nc-Spain 3 H and Nc-Spain 1 H isolates when they were analysed by the Dunn's test. Significant variations among the IRs of the isolates were also detected at 6 h pi (*P *= 0.046, Kruskal-Wallis test), although no differences were found in pair-wise analyses between the IRs of the different isolates (Figure 1C).

### Proliferation kinetics, proliferation rate determination and doubling time comparisons

**Figure 2:**
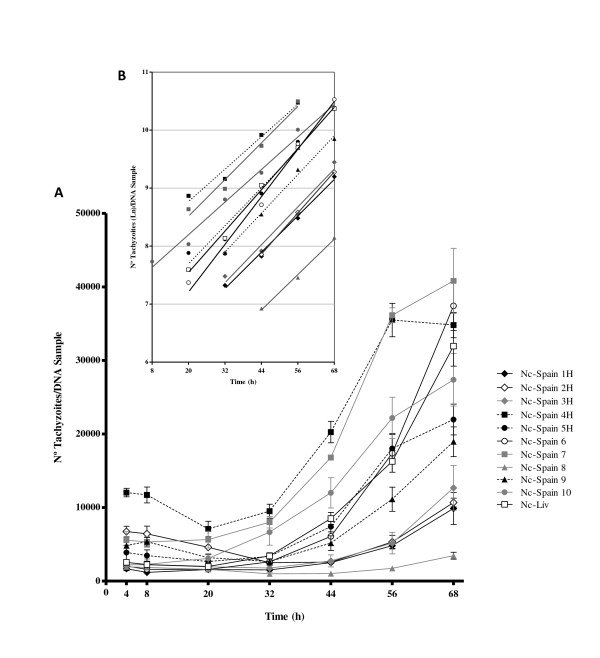
**Plot graphs representing the proliferation kinetics over time, as assessed by real-time PCR (A), and the linear regression of the average numbers of tachyzoites (ln-transformed) determined by real-time PCR against the time of the exponential phase (B) for each isolate included in this study (see graph legend)**. The average number of tachyzoites for each time in plot graphs A and B is representative of all of the individual experiments with an *R^2 ^*> 0.95, and the error bars indicate the SD. Line slopes in plot graph B define the proliferation rate (μ) and doubling time (Td, Ln 2/μ). For all isolates, *R^2 ^*> 0.98.

The parasite proliferation kinetics of each *N. caninum *isolate was studied by plotting the numbers of tachyzoites, which were determined by qPCR, against the specific collection time periods (Figure 2A). After being inoculated onto MARC-145 cell monolayers, tachyzoites did not multiply for a specific period of time, which is known as the lag phase. The lag phase of the different isolates varied between the time periods of 8 h (Nc-Spain 10), 20 h (Nc-Spain 4 H, Nc-Spain 5 H, Nc-Spain 7, Nc-Spain 6 and Nc-Liv), 32 h (Nc-Spain 1 H, Nc-Spain 3 H and Nc-Spain 9) and 44 h (Nc-Spain 2 H, Nc-Spain 8) (Figure 2A). After the lag phase, an exponential proliferation phase was observed that persisted until 56 h pi for 3 of the 11 isolates (Nc-Spain 4 H, Nc-Spain 5 H and Nc-Spain 7 isolates) and until 68 h pi for the other 8 isolates (Figure 2B). Microscopic visualisation of the inoculated cultures prior to collection verified that some parasitophorous vacuoles with tachyzoite pairs were first observed for most of the isolates at 20 h pi. After 32 h, the number of tachyzoites from the *N. caninum *isolates that were undergoing endodyogeny inside of parasite vacuoles increased with increasing time. Between 56 and 68 h pi, non-synchronous rupture of the host cells and egression of the tachyzoites were observed in the cell monolayers infected with 7 of the 11 isolates (Nc-Spain 4 H, Nc-Spain 5 H, Nc-Spain 6, Nc-Spain 7, Nc-Spain 9, Nc-Spain 10, and Nc-Liv).

**Figure 3:**
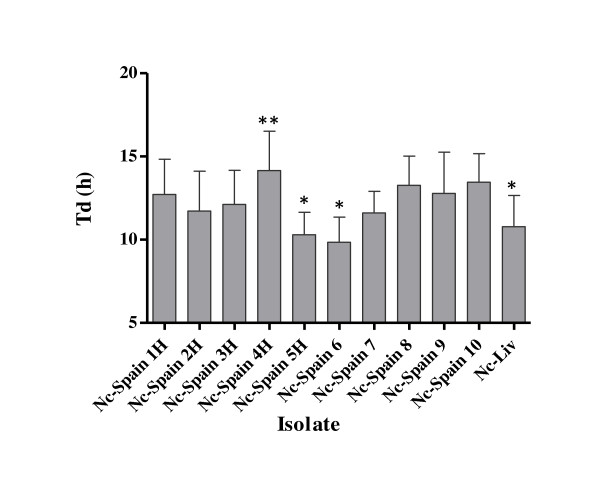
**A column graph representing the average doubling time (Td) values of replicates from experiments performed in triplicate determined in vitro for each *N. caninum *isolate**. Error bars indicate the SD. (**) marks the significantly higher Tds compared with all of those Tds that were significantly lower (*) according to the ANOVA test followed by the Tukey's test.

The μ and the Td were determined for the exponential phase, excluding the lag and egression periods, for each replicate performed for each isolate with an *R*^2 ^> 0.95. The average Td values for different isolates ranged from a minimum of 9.84 ± 1.516 h (Nc-Spain 6) to a maximum of 14.15 ± 2.364 h (Nc-Spain 4H) (Figure 3). When the Td values assessed for different isolates were compared, significant differences were detected (Figure 3). The Td value of the Nc-Spain 4 H isolate was significantly higher than the Td values of the Nc-Spain 5 H, Nc-Spain 6 and Nc-Liv isolates (*P *= 0.0016 by 1-way ANOVA, followed by Tukey's test).

### Evaluation of tachyzoite yield

**Figure 4:**
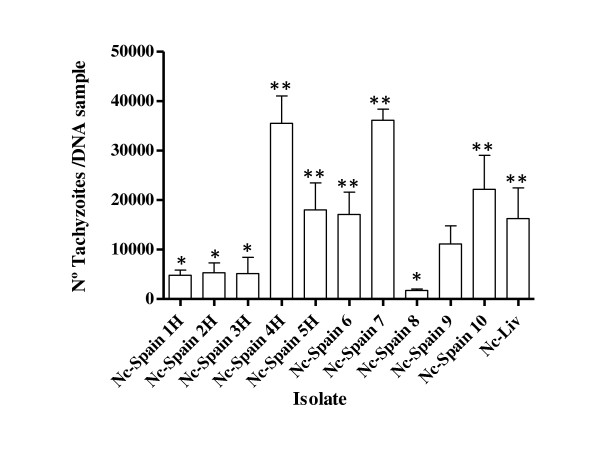
**A column graph representing the average tachyzoite yield (TY_56h_) values of replicates from experiments performed in triplicate determined in vitro for each *N. caninum *isolate**. Error bars indicate the SD. (**) marks the significantly higher TY_56 h _values compared with all those TY_56 h _values that were significantly lower (*) according to the ANOVA test followed by the Tukey's test.

The TY_56 h _was assessed to determine the number of tachyzoites produced during the same intracellular period after invasion, but prior to complete tachyzoite egression from cell monolayers. The TY_56 h _values were significantly different between isolates (*P *< 0.0001 by 1-way ANOVA, followed by Tukey's test) and varied from 1 731 (Nc-Spain 8) to 36 170 tachyzoites (Nc-Spain 7) (Figure 4). At 56 h pi, the isolates that were already undergoing exponential proliferation from 20 h pi (Nc-Spain 4 H, Nc-Spain 5 H, Nc-Spain 6, Nc-Spain 7, Nc-Spain 10 and Nc-Liv) had significantly higher TY_56 h _values than the other four isolates (Nc-Spain 1 H, Nc-Spain 2 H, Nc-Spain 3 H, and Nc-Spain 8), which began to exponentially proliferate after 20 h pi (Figure 4).

### Correlation analysis

Previous studies using a well-established pregnant BALB/c mouse model demonstrated that extensive variability exists in the pathogenicity and transmissibility of the *N. caninum *isolates included in this study [14,16]. Wide ranges in the morbidity, mortality and vertical transmission rates were observed (Table 1), suggesting a relevant role of the implicated isolate in the outcome of infection in pregnant mice. Because the in vitro IRs, Td and TY_56 h _values also varied significantly within this population of isolates, Spearman correlation analyses were applied to determine whether a potential association existed between the in vitro characteristics of isolates and their ability to be vertically transmitted and produce disease in mice. No correlations could be discerned between the Td or IR_2 h _values and vertical transmission, neonatal morbidity or neonatal mortality rates. Additionally, no correlation could be established between the vertical transmission rates and the IR_4 h, _IR_6 h _or TY_56 h _values. However, a significant correlation was found between the IR_4 h, _IR_6 h _and TY_56 h _values and neonatal morbidity and mortality rates based on the Spearman's rho coefficient (Table 2).

**Table 2:** Spearman correlation analyses of the invasion rate at 2 h pi (IR_2h_), 4 h pi (IR_4h_), 6 h pi (IR_6h_), doubling time (Td), and the tachyzoite yield at 56 h pi (TY_56h_) that were determined in vitro for each isolate in this study.

	IR_2h_		IR_4h_		IR_6h_		Td		TY_56h_	
	ρ*	*P*^#^	ρ	*P*	ρ	*P*	ρ	*P*	ρ	*P*
**Neonatal morbidity**	N.C.	-	0.7107	0.018	0.7016	0.020	N.C.	-	0.7614	0.0065
**Neonatal mortality**	N.C.	-	0.6287	0.044	0.7198	0.016	N.C.	-	0.7198	0.016
**Vertical transmission rate**	N.C.	-	N.C.	-	N.C.	-	N.C.	-	N.C.	-

* Spearman rho coefficient.

**^# ^***P *value (two-tailed).

N.C. No correlation.

## Discussion

The apicomplexan parasite *N. caninum *is an obligate intracellular parasite. The processes of parasite invasion, adaptation to new intra-cytoplasmatic conditions, intracellular proliferation, and egress from host cells constitute successive steps involved in the lytic cycle of *N. caninum *and other apicomplexa [32-34]. These processes are required for the maintenance and multiplication of the parasite in vitro and for parasite survival and propagation in the course of animal infection in vivo. As a result of a primo-infection or the reactivation of a *Neospora *infection in a chronically infected animal, tachyzoites rapidly disseminate throughout the body of the host, invade cells of different organs and cause cell death. Cell death results in the release of parasites, which develop new lytic replication cycles, thus allowing the infection to spread and cause disease [1]. Therefore, the processes involved in parasite invasion and intracellular proliferation are crucially important for understanding the pathogenesis of disease and for the development of protective vaccines and effective drug therapies. Recently, different studies have been performed to understand the precise mechanisms involved in the processes of the *N. caninum *lytic cycle, which includes parasite invasion [28,35,36] and egress [37]. However, intraspecific differences related to the efficiency of the lytic cycle processes, such as invasion and proliferation, and their association with isolate pathogenicity in vivo have been poorly investigated. The most detailed study in this regard was performed by Schock et al. [19], which revealed that differences occurred in the growth rates of six *N. caninum *isolates, although their potential correlation with virulence was not examined. Moreover, the isolates in this previous study were maintained by an undetermined number of culture passages, which could modify the original growth rate and pathogenicity of the isolates [27]. Attenuation of virulence, accompanied by faster multiplication in vitro has been previously demonstrated in *N. caninum *and *T. gondii *parasites maintained for extended periods in cell cultures, which may have been due to adaptation of the isolates to cell cultivation [27,38]. In this study, we comparatively examined the in vitro invasion efficiencies and proliferation kinetics of the active tachyzoite stage of eleven different *N. caninum *isolates, which were maintained with limited passages in cell cultures from their original isolation [5,14].

The IRs determined in this study demonstrated that the tachyzoites from most of the *N. caninum *isolates penetrated cell monolayers 2 to 4 h pi. In previous studies, tachyzoites from the Nc-1 *N. caninum *isolate penetrated bovine aorta endothelial cell cultures 45-60 min after inoculation [28]. After the initial penetrations, an insignificant increase in the number of invading tachyzoites was detected from 4 to 6 h pi. This slight increase was likely due to the loose invasion capacity of the remaining tachyzoites that had not invaded that monolayer at 4 h pi [28,35]. These results were similar to the results reported by Hemphill et al. [28], who observed that maintaining Nc-1 tachyzoites extracellularly at 37°C for time periods longer than 4-6 h resulted in decreased infectivity of the parasite. The observed differences in the invasion time periods of *N. caninum *tachyzoites may also have been influenced by the experimental conditions and the host cell types used in each experiment. *N. caninum *can be maintained in vitro in a wide variety of well-established cell cultures, and thus, this parasite can invade a wide range of cell cultures, although *N. caninum *tachyzoites from different isolates may have different affinities for specific cell types. Based on this theory, the failure to isolate parasites in CV-1 and M617 cells from a clinically affected KO mouse can be attributed to the limited invasion and growth characteristics of the specific isolate in these cell lines [39]. This theory may also explain the natural host range and the tissue tropism displayed by *N. caninum *during infection. Moreover, in previous studies, substantial differences were observed in the cell invasion processes of the closely related *N. caninum *and *T. gondii *parasites, which could explain their dissimilar host preferences [35]. However, different *N. caninum *isolates, including those that were assayed in this study, could all be adapted to the MARC-145 cell line through a limited number of cell passages [5,14,18]. Therefore, the significant differences in the invasion efficiencies observed in this study could be attributed to the biological diversity of these *N. caninum *isolates. Throughout the experiments, the IRs determined for the Nc-Spain 4 H and Nc-Liv isolates were significantly higher than the IR of the Nc-Spain 3 H isolate. Furthermore, the IRs determined for the Nc-Spain 4 H and Nc-Liv isolates were significantly higher than the IR of the Nc-Spain 1 H isolate at 4 h pi (Figure 1).

The intracellular proliferation kinetics also varied between the isolates that were analysed. Significant differences in the lag period, which ranged from 8 to 44 h pi, were observed between the isolates. Additionally, after the exponential proliferation period, the egress period, which occurred 56 to 68 h pi, was only detected in some of the isolates. Furthermore, we did not microscopically visualise parasitophorous vacuoles containing two or more tachyzoites until 20 h pi, and the cellular rupture releasing the tachyzoites was observed for some of the isolates from 56 h pi. The exponential proliferation period is delimited by the lag phase and the tachyzoite egress phase for each isolate.

The Td values, which signify the μ, also varied from approximately 10 to 14 h, and significant differences in the values were detected between the isolates. Our results were similar to the results previously obtained for the Nc-1 isolate, which displayed a lag period of 10-12 h and a Td of 14-15 h using human foreskin fibroblasts [40]. However, we must consider, as we did for the IRs, that the proliferation kinetics may have been influenced by the host cell lines used in these assays [39]. In fact, previous studies on *T. gondii *have demonstrated that differences in tachyzoite multiplication occur based on the cell lines used as hosts [41].

Variations in the IR, Td (proliferation rate) and the exponential proliferation period will determine the number of tachyzoite division cycles reached and the tachyzoite yield attained in vitro and in vivo. Significant differences in the TY_56 h _values were apparent between the isolates based on comparative analysis, and the isolates clearly grouped into two populations: "highly prolific" and "less prolific" (Figure 4). Interestingly, the severity of histopathological lesions and clinical signs have been directly related to the parasite burdens in the brains of experimentally infected mice [11,12,42-45] and to the spread of the parasite in foetal and placental tissues from infected pregnant cattle [1,15,26,32,46]. Furthermore, an association between the severity of histological lesions and parasite burdens was established in studies performed in bovine foetuses that were naturally aborted during different periods of pregnancy [47]. Therefore, the high dissemination rate, the ability to cross biological barriers (blood-brain barrier and placenta) and the enhanced invasion and proliferation rates of different *N. caninum *isolates may contribute to host tissue damage and to the severity of clinical signs in vivo. In support of this hypothesis, we observed that the isolates with the highest IRs (Nc-Spain 4 H and Nc-Liv) caused the highest morbidity in dams and the highest morbidity and mortality in neonates (100% succumbed to infection), while the isolates with the lowest IRs (Nc-Spain 1 H and Nc-Spain 3H) induced lower neonatal mortality in a pregnant mouse model [14,16]. Furthermore_, _the "highly prolific" and "less prolific" isolate populations contained isolates that displayed the highest (Nc-Spain 5 H and Nc-Spain 7) and lowest (Nc-Spain 2 H and Nc-Spain 3H) parasite burdens and the levels of histopathological lesions in the brain during the chronic phase of infection in a cerebral mouse model, respectively [12]. Interestingly, this system of ordering grouped the isolates that had the highest and lowest capacities to produce disease in the pregnant mouse model together (Table 1) [14,16]. Based on these observations, the association between the in vitro IRs and TY_56 h _values for these isolates and the neonatal morbidity, mortality and vertical transmission rates produced by these isolates in mice was investigated through a correlation analysis. A direct correlation between the IR_4 h _and IR_6 h _values when invasion was completed, as well as the TY_56 h _value and their pathogenicity in mice was established, suggesting that the in vitro invasion and proliferation rate traits are related to the in vivo virulence of the *N. caninum *isolates, at least in the pregnant BALB/c mouse model. Moreover, the Nc-Spain 1 H isolate (a "less prolific" and less invasive isolate in this study) had a limited ability to induce foetal death in a pregnant bovine model [15]. High growth rates due to a higher reinvasion capacity, but not due to significant variations between isolate-specific Tds, have also been recognised as a virulence trait in *T. gondii *[25], though pathogenesis in toxoplasmosis has mainly been found to be related to the inflammatory immune response against infection by specific *T. gondii *types [22,48]. However, virulence factors, such as the ROP18 and ROP16 rhoptry proteins, have recently been identified in *T. gondii*, and the ability of ROP18 to increase the intracellular proliferation of this parasite has been specifically suggested to cause enhanced virulence of the parasite [23,25,49]. Because the outcome of *N. caninum *infection is affected by a combination of host, parasite, and external factors, these factors need to be considered collectively when establishing direct associations between the in vitro and in vivo behaviour of *N. caninum *isolates. Various parasite factors, which include the efficacy of disseminating and crossing host barriers, may also be different between *N. caninum *isolates, as described for *T. gondii *isolate types I, II and III [22,23]. Therefore, differences may also exist between *N. caninum *isolates in the number of parasites that are able to spread throughout the host and colonise target organs (brain and placenta) and, consequently, the tachyzoite yield reached in the target tissues, the outcome of an infection and the transmission of the parasite to foetuses in pregnant animals. These differences may explain the lack of a correlation found between the IRs and TY_56 h _values with vertical transmission rates.

Interestingly, the genetically identical Nc-Spain 1 - Nc-Spain 10 and Nc-Spain 3 H - Nc-Spain 4 H isolates displayed significant differences in their in vitro behaviour, as well as in their pathogenicity in mice [14,16]. Both pairs of isolates were obtained from the same dairy herd, but from different calves, and therefore, they could be considered as different isolates that may include genetic variations in other *loci *that were not examined.

In summary, this study showed that there is intraspecific diversity in the invasion rate and proliferation kinetics of different *N. caninum *isolates. More interestingly, the correlation found between the in vitro characteristics of the isolates with their in vivo pathogenicity in pregnant mice and their offspring confirms that invasion and proliferation rates are virulence traits in *N. caninum*. Within apicomplexan parasites, host cell invasion and intracellular proliferation are tightly regulated processes that involve the sequential secretion of components from specialised organelles (micronemes, rhoptries and dense granules). A large number of these secreted elements and their interactions have already been identified in *T. gondii *[24]. Some of these elements, such as the ROP18 and ROP16 proteins, are virulence factors in *T. gondii *[23,25,49]. In contrast to *T. gondii*, the role of orthologous microneme, roptry and dense granule proteins in *N. caninum *is still unclear. In addition, cell culture-based approaches have demonstrated significant differences between *T. gondii *and *N. caninum *species [32,35]. Further studies are necessary to identify the molecular mechanisms within *N. caninum *that are directly involved in mediating the differences observed between various isolates.

## Competing interests

The authors declare that they have no competing interests.

## Authors' contributions

JRC carried out in vitro assays, conceived the study, participated in its design, and drafted the manuscript. MGB participated in invasion assays, in its design and helped to draft the manuscript. IS performed invasion and proliferation assays. GA participated in the design of the study and coordination. GAG participated in proliferation assays and performed the statistical analysis. IP carried out real-time PCR determinations. LMO also conceived the study, participated in the design of the study and coordination, and helped to draft the manuscript. All authors read and approved the final manuscript.
